# Effect of social media–based diabetes self-management education and support on glycemic control in adults with type 2 diabetes: a systematic review and meta-analysis

**DOI:** 10.3389/fcdhc.2026.1907185

**Published:** 2026-10-28

**Authors:** Lisa Diaz, Dante Anthony Tolentino, Evelyn Scott, Mary-Lynn Brecht, Wendie Robbins, Sarah Choi

**Affiliations:** 1 School of Nursing, University of California, Los Angeles, Los Angeles, CA, United States; 2 Pomona Valley Hospital Medical Center, Pomona, CA, United States

**Keywords:** diabetes mellitus, DSME, HgbA1c, meta - analysis, social media, systematic review, type 2 diabetes

## Abstract

**Background:**

Digital interventions are being used for diabetes self-management education and support (DSMES), but evidence of their effectiveness is limited. This review and meta-analysis examined the impact of social media-based DSMES on glycemic control in adults with type 2 diabetes (T2DM), and an exploratory *post hoc* analysis assessed the effect of intervention duration on outcomes.

**Methods:**

A PRISMA 2020-compliant systematic review was conducted using PubMed, CINAHL, Web of Science, and PsycINFO, with 789 records screened and one additional record identified through a saved search (total of 790). Eligible studies were English-language articles published over a just-over-ten-year period. These studies focused on adults with T2DM, reported HbA1c outcomes, and utilized social media for DSMES interventions. Risk of bias was assessed using the Cochrane Risk of Bias 2 (RoB 2) tool for randomized controlled trials (RCTs). A random-effects meta-analysis estimated pooled mean differences (MD) in HbA1c percentage points, with subgroup analyses by intervention duration; heterogeneity was assessed using the I² statistic.

**Results:**

This systematic review identified 13 studies, including 11 RCTs for the meta-analysis. The pooled analysis demonstrated a statistically significant overall effect favoring the intervention (MD = −0.44 HbA1c percentage points, 95% CI −0.87 to −0.00, *p* = 0.05). Substantial between-study heterogeneity was observed (I² = 91%), and the 95% prediction interval ranged from -2.08 to 1.20. Interventions of 3 months or less showed no significant effect (MD = -0.12, 95%CI -0.51 to 0.28, *p* = .56), whereas interventions longer than three months showed a large, statistically significant effect (MD = −1.02, 95% CI −1.75 to −0.29, *p* = 0.01). The test for between-subgroup differences was significant (Q = 4.56, df = 1, *p* = .03).

**Conclusion:**

Social media-based DSMES interventions may be associated with improvements in glycemic outcomes in adults with T2DM. While interventions longer than three months showed greater pooled effects, interpret these *post hoc* results carefully because of significant heterogeneity (I² = 91%). This pooled estimate represents an average effect across heterogeneous interventions and populations and therefore should not be understood as a uniform effect of social media-based DSMES.

**Systematic review registration:**

https://doi.org/10.17605/OSF.IO/BZR6P, OSF Registries BZR6P.

## Background

Type 2 Diabetes Mellitus (T2DM) continues to be a global health challenge, underscoring the need for innovative self-management strategies to manage this complex metabolic condition effectively. T2DM is characterized primarily by persistently elevated blood glucose levels, a condition referred to as hyperglycemia (1). The condition often arises from a progressive deterioration in the pancreas’s capacity to synthesize adequate quantities of insulin, and this decline is frequently exacerbated by insulin resistance, characterized by a reduced sensitivity of the body’s tissues to the actions of insulin (2). According to the World Health Organization (WHO), persistent hyperglycemia contributes significantly to microvascular and macrovascular complications, adversely affecting cardiovascular health, renal function, neuropathy, retinopathy, and overall metabolic regulation (3).

Hemoglobin A1c (HbA1c) is widely recognized as the “gold standard” for guiding the clinical management of diabetes mellitus (4). A 0.5% reduction in HbA1c is considered clinically significant, as research shows that better glycemic control is associated with improved outcomes (5–7). A 1% decrease in HbA1c was associated with a 35% reduction in diabetes-related complications, according to the United Kingdom Prospective Diabetes Study (8). Furthermore, research indicates that HbA1c values exceeding 10% are associated with higher healthcare costs than those below 6% (9). Glycemic control is associated with lower total healthcare costs (10). Addressing self-care, self-efficacy, and health literacy is also important for diabetes management. Self-care encompasses various dimensions and involves a range of actions (e.g., monitoring blood glucose levels, diet management, and exercise) (11). Research shows that self-efficacy is crucial for changing health behaviors, thereby improving self-management of chronic illnesses such as diabetes (12). Health literacy, defined as the ability to obtain, comprehend, and apply health-related knowledge and exercise appropriate judgment, plays a significant role in patient management (13).

For individuals with diabetes, Diabetes Self-Management Education and Support (DSMES) programs are crucial for improving glycemic control and self-care behaviors, with multifaceted benefits encompassing improvements in clinical, psychosocial, and behavioral outcomes (14, 15). However, participation is often limited by cultural and language barriers, accessibility, time constraints, service shortages, and geographic barriers (16). In certain Asian communities, the impact of stigma and shame associated with diabetes reduced their openness to participating in organized DSMES programs (17). Ultimately, diabetes self-management predominantly occurs outside of clinical settings, placing the responsibility for self-care on people living with diabetes (and their caregivers) (18).

Promoting access to DSMES services by recognizing and addressing population barriers, as well as health inequalities, is crucial (19). The rapid growth of digital technologies, including smartphone applications, wearable devices, sensors, and online health networks, has enabled more scalable and personalized approaches to behavior-change interventions (20). The increased use of these technologies is driving changes in diabetes management, a shift accelerated by the COVID-19 pandemic (21). Digital technologies can facilitate the delivery of healthcare, consultations, and clinical services, as well as ongoing patient monitoring beyond in-person care (22). Among digital technologies, social media platforms have become a prominent source of medical information, including information on diseases and personal health experiences (23). The same authors also noted that social media creates new avenues for people to share their experiences and is increasingly significant in spreading public health messages. In one study, the authors proposed that social media could be a beneficial tool for improving self-management among older adults with T2DM when used in moderation, with no more than 139.8 minutes per day (24). Social media platforms like Twitter, YouTube, TikTok, Facebook, Snapchat, Reddit, Instagram, and WhatsApp provide online spaces for individuals to interact and exchange information (25). Additionally, social media platforms have become essential for healthcare organizations to engage the public and promote prevention and disease management, particularly in under-resourced areas (26).

Several recent studies indicate that more individuals are seeking diabetes-specific information, education, and support through social media (27–31). Access to digital technologies for T2DM is proving vital, especially in rural areas with limited healthcare access and in populations with high diabetes prevalence (32). Despite this trend, evidence regarding the clinical effectiveness of social media–based DSMES interventions in diverse populations with T2DM remains limited. This systematic review and meta-analysis aimed to assess the effectiveness of social media-based DSMES interventions on glycemic control, as measured by HbA1c, in adults with T2DM, stratified by intervention duration. Secondary outcomes of interest included diabetes self-care, efficacy, and health literacy.

For this review, social media-based DSMES was defined as existing platforms that allow users to generate, receive, and interact with DSMES content through individual and group communication, multimedia sharing, and social networking (33). Qualifying studies used established social media platforms as the primary means of distributing DSMES content. Previous researchers have identified platforms such as Facebook, WeChat, WhatsApp, LINE, and Telegram, which facilitate interactive communication among participants (34).

### Conceptual framework

This systematic review was conceptually grounded in Social Cognitive Theory (SCT), which posits that health behavior change results from the dynamic of intrapersonal influences, behavioral capabilities, and environmental forces (35). Central to SCT are constructs such as self-efficacy, outcome expectations, observational learning (modeling), reinforcement, and self-regulation, all of which are highly relevant to diabetes self-management behaviors. Prior integrative research has demonstrated that health promotion campaigns and social media–based interventions frequently employ SCT as a guiding framework for behavior change (36, 37).

## Methods

### Protocol and registration

The protocol for this systematic review and meta-analysis was retrospectively registered on the Open Science Framework (OSF) (https://doi.org/10.17605/OSF.IO/BZR6P) on January 19, 2026. This registration occurred one month after title and abstract screening began because of a delay in submission on OSF. The review protocol, including the eligibility criteria, search strategy, and planned analyses, was finalized before screening and remained unchanged after registration. A timestamped version of the original protocol is publicly accessible via the OSF record.

### Eligibility criteria

This study included Randomized Controlled Trials (RCTs) and quasi-experimental studies with pre- and post-HbA1c data that met the PICOT framework. This review included all international studies of social media DSMES interventions, in any language, that were accessible in English. Only full-text articles published in English from peer-reviewed journals were included due to translation resource constraints.

Population (P): Studies conducted in any country that included adults (aged 18 years or older) diagnosed with type 2 diabetes.

Intervention (I): Interventions that provided evidence-based DSMES via social media platforms and conducted pre- and post-intervention assessments.

Comparator (C): Included usual care, alternative education delivery modalities, waitlist control, or no intervention. Single-arm pre-post studies with baseline and follow-up HbA1c values were eligible for the systematic review but not for the meta-analysis, which was restricted to RCTs with a comparator group.

Outcomes (O): The primary outcome was post-intervention HbA1c. Secondary outcomes included evaluations of diabetes self-care, efficacy, and participants’ health literacy.

Time Frame and Publication Characteristics: Published between 2015 and February 2026 in peer-reviewed journals and available in English. This timeframe facilitated examination of foundational studies that continue to influence contemporary practices (38). This approach also enables analysis of technological evolution in the field, highlighting significant advancements and shifts over the past decade.

### Exclusion criteria

Studies were excluded if they: 1) evaluated digital interventions delivered through non-social media applications, 2) focused on diabetes blogging, do-it-yourself (DIY) diabetes information, or informal peer support without structured DSMES, 3) were conducted exclusively in T1D or gestational diabetes, or 4) enrolled participants younger than 18 years. Conference abstracts, gray literature, dissertations, theses, and preprints were excluded from this review. Gray literature (e.g., dissertations, reports, conference abstracts) was excluded due to the potential for insufficient methodological transparency and reporting. Interventions delivered solely through websites, text messaging, mobile health applications without social networking functionality, telehealth videoconferencing, or email were excluded.

### Information sources

The search was conducted across multiple bibliographic databases, including PubMed, Web of Science, Cumulative Index to Nursing and Allied Health Literature (CINAHL), and PsycINFO. An initial search of all four databases was conducted on December 10, 2025, covering the period from January 2015. Ongoing monitoring of newly published records continued using the PubMed advanced search builder and saved search functionality. This automated PubMed search identified one additional eligible record in February 2026: Zheng et al., 2026 (38), which was added to the review.

### Search strategy

This systematic review and meta-analysis was conducted in accordance with the Cochrane Handbook for Systematic Reviews of Interventions (39) and the 24-step guide on conducting and publishing systematic reviews and meta-analyses in medical research (40). We then used the Preferred Reporting Items for Systematic Reviews and Meta-Analyses (PRISMA) guidelines to report results (41).

This search utilized specific keywords, database-specific controlled vocabulary, Medical Subject Headings (MeSH) terms, and Boolean combinations tailored to each specific database. The queries included keywords, phrases, synonyms, and indexing terms as follows: “diabetes self-management education,” OR “diabetes self-management education support,” OR “DSME,” OR “diabetes knowledge,” OR “diabetes training,” OR “DSMES,” OR “diabetes education,” OR “chronic disease education,” OR “self-management,” and “social media,” OR “social networks.” See **Supplementary Material** for the full reproducible search strategy for each database.

### Study selection

All studies were imported and assessed using Covidence^®^, a web-based software platform for systematic and literature reviews (42). The software removed duplicates before screening. Two independent reviewers (L.D. and E.S.) screened titles and abstracts for eligibility and compared lists to resolve disagreements. A third reviewer (D.T.) resolved any discrepancies. L.D. and E.S. performed full-text reviews of the final study articles.

### Data extraction

Data extracted included country, language, study design, social media platform, sample size, intervention duration, intervention components, behavioral theory, participant characteristics (age, sex, education), HbA1c outcomes, and results of diabetes self-care assessments, diabetes knowledge, and health literacy. The study’s authors were contacted for additional data as needed and notified of this analysis. EndNote reference management software was used for citation and PDF management; only full-text articles were included.

### Risk of bias assessment

The Cochrane Risk of Bias 2 (RoB 2) tool was used to assess risk of bias in the randomized controlled trials, which assesses biases across five domains:1) Bias arising from the randomization process, 2) Bias due to deviations from intended interventions, 3) Bias stemming from missing outcome data, 4) Bias in the measurement of outcomes, and 5) Bias in the selection of reported results. Each domain and overall risk were categorized as “low risk of bias,” “some concerns,” or “high risk of bias” (43). Quasi-experimental studies were assessed using the Risk of Bias In Non-randomized Studies of Interventions (ROBINS-I) tool (44), with each study judged across seven domains: confounding, participant selection, classification of interventions, deviations from intended interventions, missing data, outcome measurement, and selective reporting, and assigned an overall risk of bias rating (low, moderate, serious, critical) based on these assessments. Two independent reviewers (L.D. and E.S.) conducted all assessments. Reviewers addressed discrepancies through discussion, and consulted a third reviewer (D.T.) when they could not reach consensus. Risk-of-bias assessments were incorporated into the interpretation of the findings.

### Effect measures

All included trials reported HbA1c as a percentage. Five trials stated the unit explicitly (45–49) while the remaining six trials did not include the unit (31, 50–54). No trial reported HbA1c in mmol/mol, and no unit conversion was required.

The primary effect measure was the mean difference (MD) in HbA1c percentage points (%) between intervention and control groups. Because HbA1c is measured on a single, clinically interpretable scale across all trials, the unstandardized mean difference was used rather than a standardized measure (55).

Effect sizes were computed from post-intervention means and SDs for each arm, based on the number of participants analyzed at follow-up. Where trials reported multiple follow-up timepoints, outcomes were extracted at the final reported HbA1c assessment.

Two trials required deviations. Kim et al. reported HbA1c only within insulin-treatment strata, as estimated marginal means with SE (52). Whole-arm means and SD were reconstructed by combining strata using standard formulas (56), with stratum sizes taken from the trial’s **Supplementary Tables**. This method was validated by reproducing the trial’s independently reported whole-arm baseline values. Shi et al. reported change-from-baseline estimates rather than post-intervention means (47). Post-intervention values were derived by applying the reported 6-month change estimates to the reported baseline means, with baseline SDs carried forward. An alternative derivation of SDs was using the trial’s reported CI; however, this only changed the pooled estimate by < 0.01%. Where SDs were not reported, they were derived from reported standard errors (SEs) using the formula SD = SE × √n (57). Between-study variance was estimated by restricted maximum likelihood. All analyses were conducted in IBM SPSS Statistics Version 31.0.1.0 (49).

### Synthesis methods

Random-effects meta-analysis was conducted to account for expected clinical and methodological heterogeneity. Eligibility for the meta-analysis was restricted to randomized controlled trials (RCTs). Quasi-experimental studies lacking a comparator group were excluded from the meta-analysis. Statistical heterogeneity was assessed using the I² statistic. To explore whether intervention duration was associated with differences in HbA1c outcomes, interventions were divided into those with a duration of ≤ 3 months and those with a duration of > 3 months for subgroup analyses. This timeframe was also utilized in a prior meta-analysis on DSMES, which investigated the impact of intervention duration in subgroup analyses on changes in HbA1c (58, 59). Researchers frequently assess HbA1c at baseline, 3 months, and 6 months (60, 61) because these time points align with HbA1c’s measurement utility. For secondary outcomes not included in the meta-analysis, a narrative synthesis was employed. Notably, the authors used diverse outcome measurement instruments and analytic methods in measuring secondary outcomes. Between-study variance was estimated by restricted maximum likelihood, and differences between duration subgroups were tested using a between-subgroup homogeneity test.

### Exploration of heterogeneity

Given the potential variability among the studies, additional sources of heterogeneity were assessed descriptively. Factors considered included the type of social media platform, intervention components, duration, healthcare professional involvement, baseline HbA1c levels, geographic location, study design, and risk of bias. As a result, exploratory *post hoc* analyses focused on intervention duration, while other factors were assessed through narrative examination.

### Sensitivity analysis

A sensitivity analysis was conducted to evaluate the stability and reliability of the pooled effect estimate and to determine whether any single study disproportionately influenced the overall findings. Specifically, a leave-one-out approach was applied, in which each study was sequentially removed to assess its impact on the overall effect size and between-study heterogeneity. Meta-regression was considered; however, a general recommendation suggests having at least 10 high-quality studies with minimal bias (62). Because this meta-analytic review included only 11 small studies and the reported intervention characteristics were inconsistent, meaningful analyses were considered not possible. To assess publication bias and small-study effects, a funnel plot was inspected, along with Egger’s test (63) and the trim-and-fill procedure was used to estimate missing studies.

## Results

A systematic search across four databases (PubMed, CINAHL, Web of Science, and PsycINFO) initially yielded 789 records, and one additional record was identified via a PubMed saved search builder. Prior to screening, 81 duplicates were removed from the database records, leaving 708 records to be screened by title and abstract. Of these, 671 records were excluded. A total of 37 full-text studies, together with the one additional record identified through the saved search, were assessed for eligibility. Of the 38 records assessed, 25 were excluded (wrong setting, type of diabetes, intervention, time period, study design, and population), and 13 studies that utilized social media applications to implement DSMES interventions aimed at individuals with T2DM were selected (31, 45–54, 64, 65). See **Figure 1** for the Preferred Reporting Items for Systematic Reviews and Meta-Analyses (PRISMA) flow diagram below (41).

**Figure 1:**
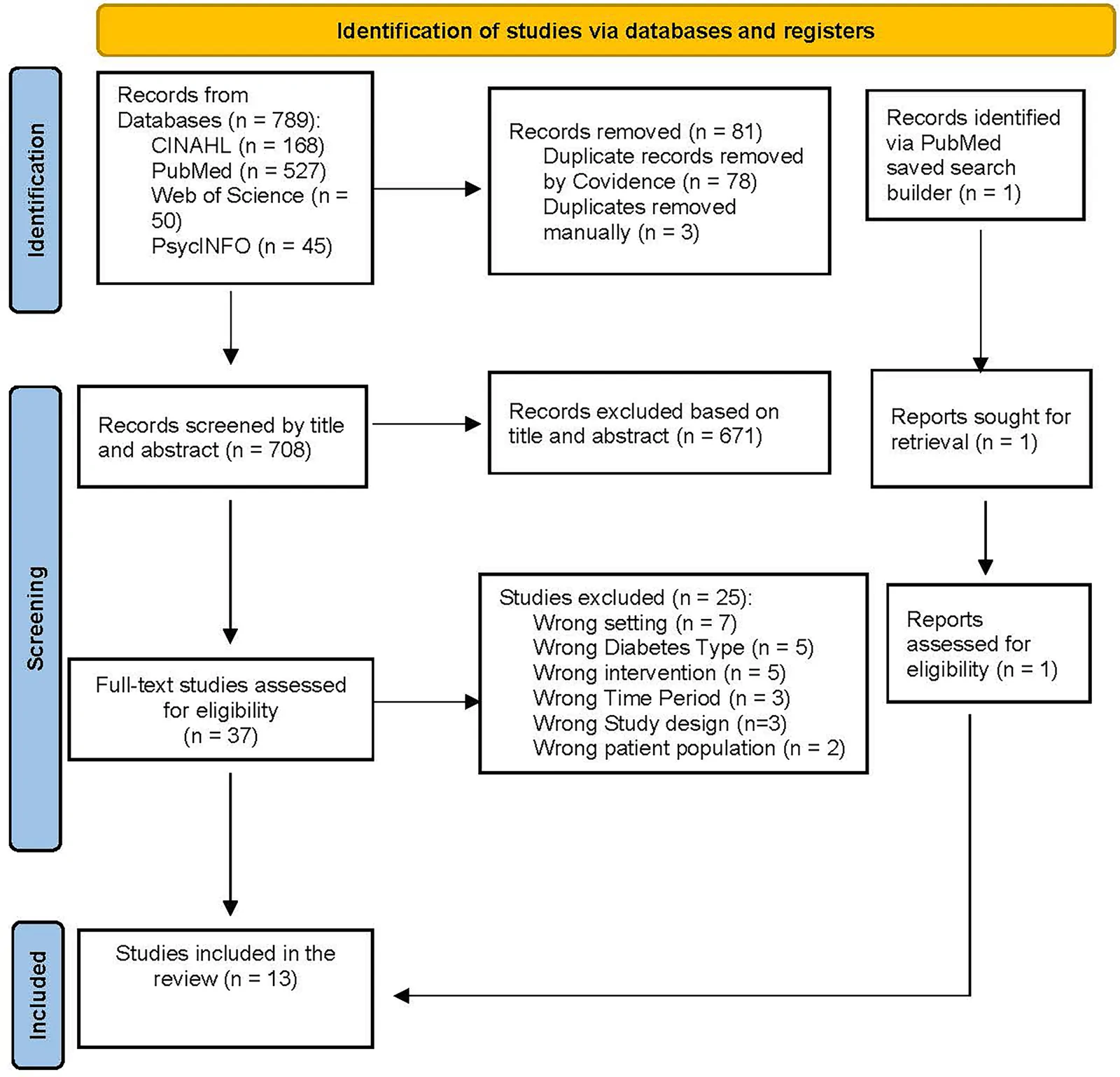
PRISMA flow chart of search strategy and study selection.

Most publications (75%) were produced in the last five years. In terms of study design, a total of 11 studies were RCTs (31, 45–54), while two were considered quasi-experimental studies with single-arm pre- and post- test designs (64, 65).

Research spanned a diverse range of geographical regions, including high- and middle-income countries, with a primary emphasis on urban environments. Several studies specifically targeted low-income participants or addressed the needs of remote populations. The countries involved in this research included China (n = 3), the United States (n = 2), Iran (n = 2), Saudi Arabia (n = 2), South Korea (n = 2), as well as one study each from Taiwan and the United Arab Emirates. The following distribution of languages and social media platform adoption was observed: six studies conducted interventions in Chinese, with four studies utilizing WeChat (45, 47, 49, 51, 65) and one utilizing LINE (48). Additionally, three studies implemented interventions in Arabic via WhatsApp (31, 46, 64). Two other studies conducted interventions in Korean on Naver Café (49, 50), and the final two studies conducted their investigations in Farsi and used Telegram/Soroush (48, 52); see **Table 1**. No studies were conducted in English or Spanish; the U.S. studies recruited Chinese-speaking participants.

**Table 1:** Characteristics of included social media-based studies.

			Study							Participants		
Author (country)	Year	Language	Social-media platform	Design	Sample size (N)	Intervention duration (months)	Frequency of interaction	Facilitator/educator type	Outcome measures	Age (years, mean)	Education less than high school (%)	Gender (%)
Al Omar et al. (United Arab Emirates)	2020	Arabic	WhatsApp	RCT	164	6.0	Group messages; bidirectional.Unknown frequency.	Licensed pharmacists	HbA1c; LAD; DNT	41.98	8.40	Male = 42.1
Alhazmy et al. (Saudi Arabia)	2024	Arabic	WhatsApp	Quasi-experimental	62	3.0	Daily group messages, group Q&A.	Registered nurse, diabetes educator, and medical–surgical nursing professors	HbA1c; Diabetes Self-Care Scale	47.60	58.10	Male = 0
Dong et al. (China)	2018	Chinese	WeChat	RCT	120	12.0	Weekly educational sessions	Trained nurses (college degree+, 3+ years experience)	HbA1c; Diabetes Management Self-Efficacy; BMI; FBG	42.70	56.66	Male = 52.1
Hu et al., (United States)	2022	Chinese	WeChat	Quasi-experimental	30	3.0	2 videos per week	Bilingual study staff (reviewed by certified diabetes educator and nurse)	HbA1c; Stanford 8-item Self-Efficacy for Diabetes scale; IPAQ	61.00	63.00	Male = 30.0
Kargarshuroki et al. (Iran)	2023	Farsi	Telegram/Soroush	RCT	134	1.5	Daily messages, inspirational Friday messages, and holiday messages.	Research team (supported by endocrinologists, dietitians, and health educators)	HbA1c; IPAQ; diabetes awareness questionnaire; Lipid profile	56.39	73.00	Male = 48.5
Kim & Utz, (South Korea)	2019	Korean	Naver Band	RCT	151	2.0	Group messages 1–2 per week	Nurse with 5+ years of diabetes education experience	HbA1c; SDSCA; PAM13-K; HL	51.33	29.67	Male = 45.7
Kim et al. (South Korea)	2022	Korean	Naver Café	RCT	89	6.0	Group forums, weekly polls	Diabetes nurse educators	HbA1c; SDSCA; FBG; triglyceride; total cholesterol	49.34	15.70	Male = 51.7
Leong et al., (Taiwan)	2022	Chinese	LINE	RCT	181	3.0	Group messages 2 per week	Pharmacists	HbA1c; SDSCA; SDKS; DCP-ATDS; HL	58.60	15.00	Male = 68.5
Safdari et al., (Iran)	2025	Farsi	Telegram	RCT	70	1.0	Daily messages	Lead researcher	HbA1c; Self-care questionnaire; HELIA	46.40	54.33	Male = 28.3
Shi et al. (United States)	2025	Chinese	WeChat	RCT	60	3.0	2 pre-recorded videos per week	Research Team	HbA1c; BMI; IPAQ; Mediterranean Dietary Screener	54.30	18.75	Male = 61.7
Xia et al., (China)	2022	Chinese	WeChat	RCT	120	6.0	Weekly messages	Multidisciplinary team (general physician, specialist nurse, endocrinologists, dietitian)	HbA1c; BMI; BP; Lipid profile; FBG	60.04	60.00	Male = 63.3
Yaagoob et al., (Saudi Arabia)	2024	Arabic	WhatsApp	RCT	80	1.5	Weekly messages; weekly and facilitated discussion	Access to a diabetes nurse and diabetologist access	HbA1c; DSMES-UK; DKT2; HADS; DQoL	40.55	14.90	Male = 65.0
Zheng et al., (China)	2026	Chinese	WeChat	RCT	556	3.0	3 modules & tasks per week	Multidisciplinary team (family physicians, online health assistants, specialists)	HbA1c; BMI; BP; Lipid profile; FBG	59.10	47.12	Male = 40.9

RCT, randomized controlled trial; Quasi-exp., quasi-experimental; I, Intervention, C, Control; *, No Control; HbA1c, hemoglobin A1c; LAD, Literacy Assessment of Diabetes; DNT, Diabetes Numeracy Test; BMI, Body Mass Index; FBG, Fasting blood glucose; SDSCA, Summary of Diabetes Self-Care Activities; SDKS, Simplified Diabetes Knowledge Scale; DCP-ATDS, Diabetes Care Profile–Attitudes Toward Diabetes Scales; IPAQ, International Physical Activity Questionnaire; PAM13-K, Patient Activation Measure 13 Koren Version; HL, health literacy; BMI, body mass index; BP, blood pressure; HELIA, Health Literacy for Iranian Adults; DSMES-UK, UK-Diabetes management Self-Efficacy scale; DKT2, Diabetes Knowledge Test 2; HADS, Hospital Anxiety and Depression Scale; DQoL, Diabetes quality of lifePublication bias.

### Participant characteristics

A total of 1,817 participants were enrolled across the 13 studies; enrollment ranged from 30 to 556, with an average of 120 participants. Overall, male and female participation was nearly equal (50%), although one small study included only female participants (63) and another had a 2:1 male-to-female ratio (48). All studies reported educational attainment; half reported that more than 42% of participants had not completed high school. The percentage of participants with less than a high school education ranged from 8.4% to 73.0%, with a mean of 39.59%. All studies reported age; the mean age was 51.49 years, with study-level averages ranging from 40.55 to 61.00 years. See **Table 1** for study and participant characteristics.

Any publication bias was assessed on the primary mean difference scale. Visual inspection of the funnel plot did not indicate meaningful asymmetry, and Egger’s regression-based test was not significant (coefficient = -.016, SE = 3.34, t = -0.05, *p* = .96, 95%CI -7.72 to 7.40) See **Figure 2**. A trim-and-fill analysis imputed no missing studies, leaving the pooled estimate unchanged. These results should not be interpreted as evidence that publication bias is absent. With only 11 trials and substantial heterogeneity (I² = 91%), both funnel plot inspection and Egger’s test have limited power, and publication bias cannot be excluded (66).

**Figure 2:**
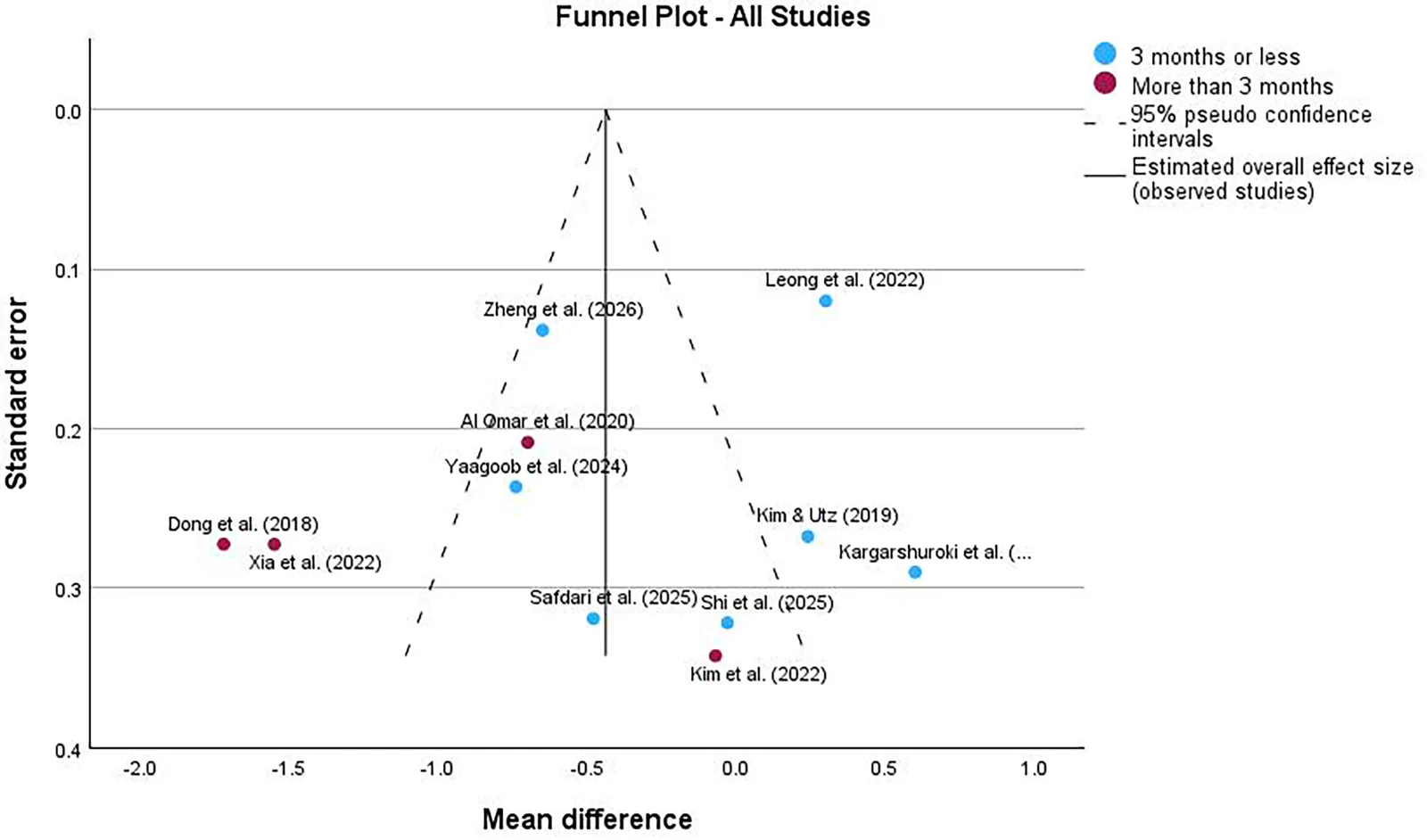
Funnel plot of RCT studies.

### Risk of bias

The risk-of-bias assessment showed that four randomized controlled trials had a low risk of bias, while seven had some concerns according to the RoB 2 tool; no trials were classified as high risk. Common concerns included issues with randomization reporting and the selection of reported results. Bias from missing outcome data and measurement error was generally low (**Figure 3**).

**Figure 3:**
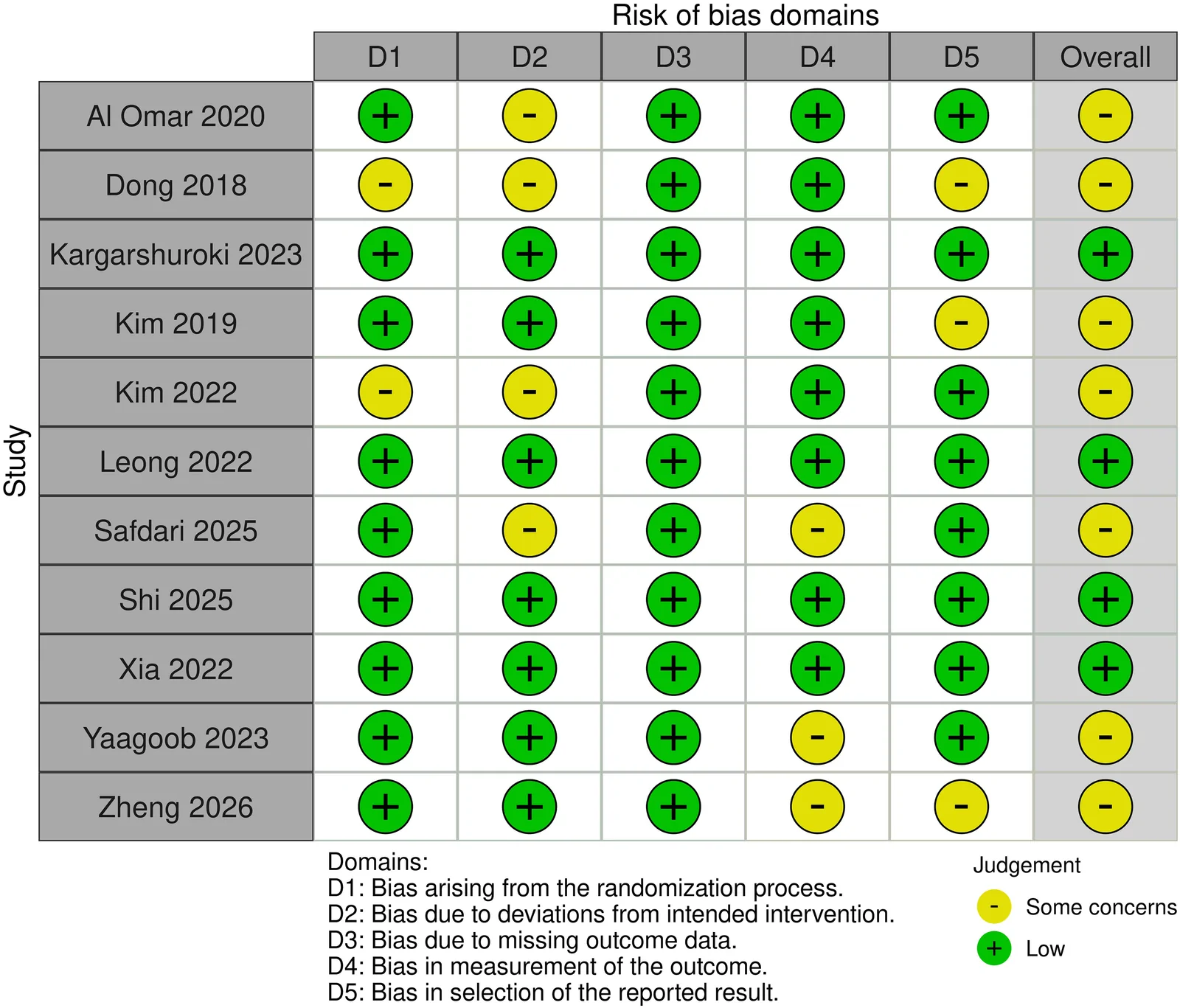
Risk of bias domains summary.

In contrast, two quasi-experimental studies assessed with ROBINS-I were deemed to have a serious risk of bias due to confounding and the lack of comparator groups (**Figure 4**). Overall, the evidence quality was moderate, though variations in study quality may have contributed to between-study heterogeneity.

**Figure 4:**
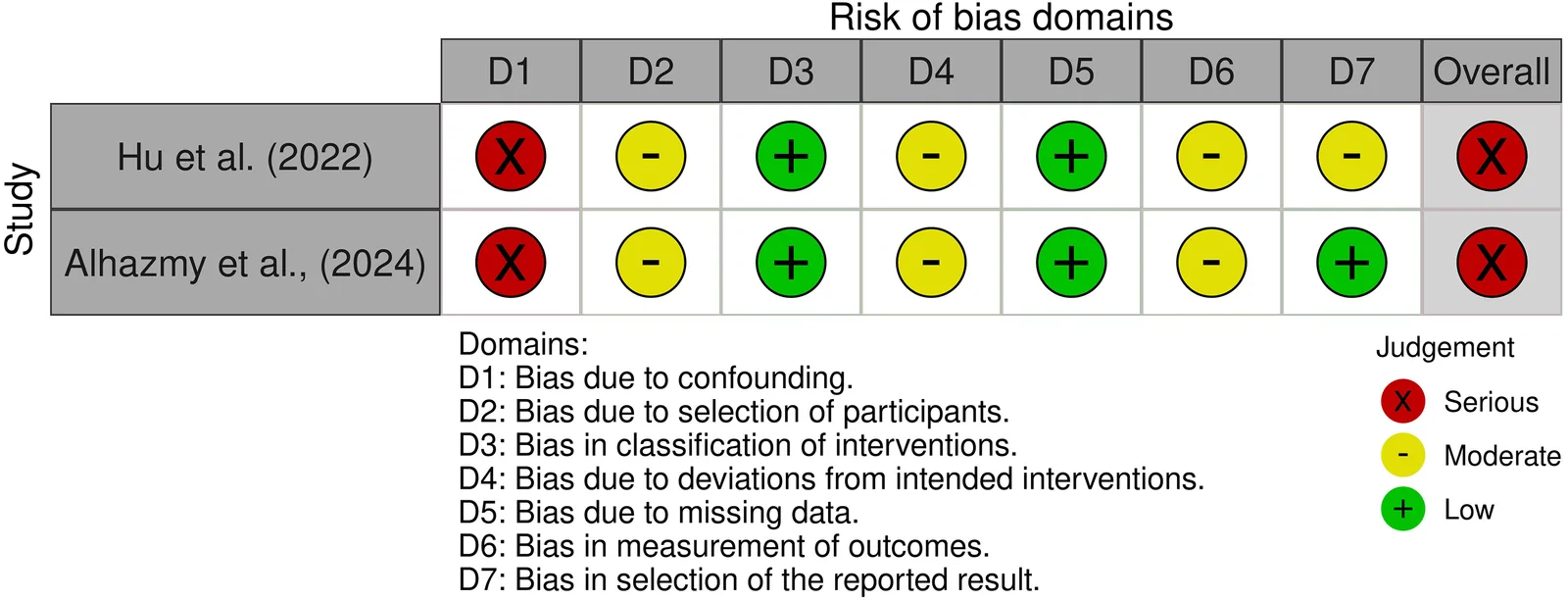
Risk of bias in nonrandomized studies of interventions summary.

### Changes in HbA1c

Descriptive statistics from the 13 studies showed improved glycemic control. Across intervention arms, baseline HbA1c averaged 8.47% (SD = 1.01) and post-intervention HbA1c averaged 7.47% (SD = 0.75), a reduction of about 1%. Reported changes varied substantially across studies, ranging from minimal change to reductions exceeding two percentage points (see **Table 2**). Control arms in the 11 RCTs also improved by an average of 0.47%, declining in 8 of 11 trials. These uncontrolled study-level averages are descriptive only and do not estimate a treatment effect. The controlled between-group estimate is reported below.

**Table 2:** Changes in hemoglobin A1c between intervention and control groups.

	Intervention group		Control group	
Author(s) (Year)	Baseline HbA1c % (SD)	Post HbA1c % (SD)	Baseline HbA1c % (SD)	Post HbA1c % (SD)
Al Omar et al. (2020) (46)	8.40 (1.06)	7.70 (1.35)	8.50 (1.29)	8.40 (1.32)
Alhazmy et al. (2024) (64)	8.61 (1.70)	7.92 (1.60)	*	*
Dong et al. (2018) (51)	9.55 (2.38)	6.63 (1.17)	9.23 (2.13)	8.35 (1.75)
Hu et al. (2022) (65)	7.30 (1.30)	6.90 (1.30)	*	*
Kargarshuroki et al. (2023) (54)	8.19 (2.10)	8.05 (1.96)	7.53 (1.67)	7.45 (1.34)
Kim & Utz (2019) (53)	9.55 (1.66)	6.98 (1.36)	8.73 (1.43)	6.74 (1.07)
Kim et al. (2022)^a^ (52)	9.38 (1.99)	7.48 (1.97)	9.09 (2.08)	7.57 (1.60)
Leong et al. (2022) (48)	6.90 (0.80)	7.00 (0.90)	6.70 (0.60)	6.70 (0.70)
Safdari et al. (2025) (50)	7.65 (1.42)	7.45 (1.34)	7.93 (1.46)	7.93 (1.33)
Shi et al. (2025)^b^ (47)	8.35 (1.30)	7.27 (1.30)	8.12 (1.19)	7.30 (1.19)
Xia et al. (2022) (45)	8.50 (2.13)	6.87 (1.11)	8.58 (2.02)	8.42 (1.83)
Yaagoob et al. (2024) (31)	10.31 (1.30)	9.52 (0.98)	10.41 (1.38)	10.26 (1.13)
Zheng et al. (2026) (49)	7.39 (1.53)	7.35 (1.49)	7.44 (1.46)	8.00 (1.76)

Standard deviations are presented in parentheses; HbA1c, hemoglobin A1c; *, no control group.

a Authors reported HbA1c only within insulin-treatment strata, as estimated marginal means with SEs. Whole-arm post-intervention was derived by combining strata. Baseline values are as reported for the whole arms in the **Supplementary Material**.

b Post-intervention values derived from reported change estimates; baseline SDs carried forward.

### Meta-analysis with subgroup analyses

Of the 13 studies in this systematic review, 11 were included in the meta-analysis. The pooled analysis demonstrated a statistically significant overall effect favoring the intervention (MD = -0.44, SE = 0.22, 95% CI -0.87 to -0.00, z = -1.97, *p* = .05). Substantial between-study heterogeneity was observed (τ^2^ = 0.48, H^2^ = 10.75, I^2^ = 91%; Q = 101.63, df = 10, *p* <.001). Without the subgroup analysis, the overall 95% prediction interval ranged from -2.08 to 1.20, indicating that the effect in a future trial could plausibly favor either group. See **Figure 5**.

**Figure 5:**
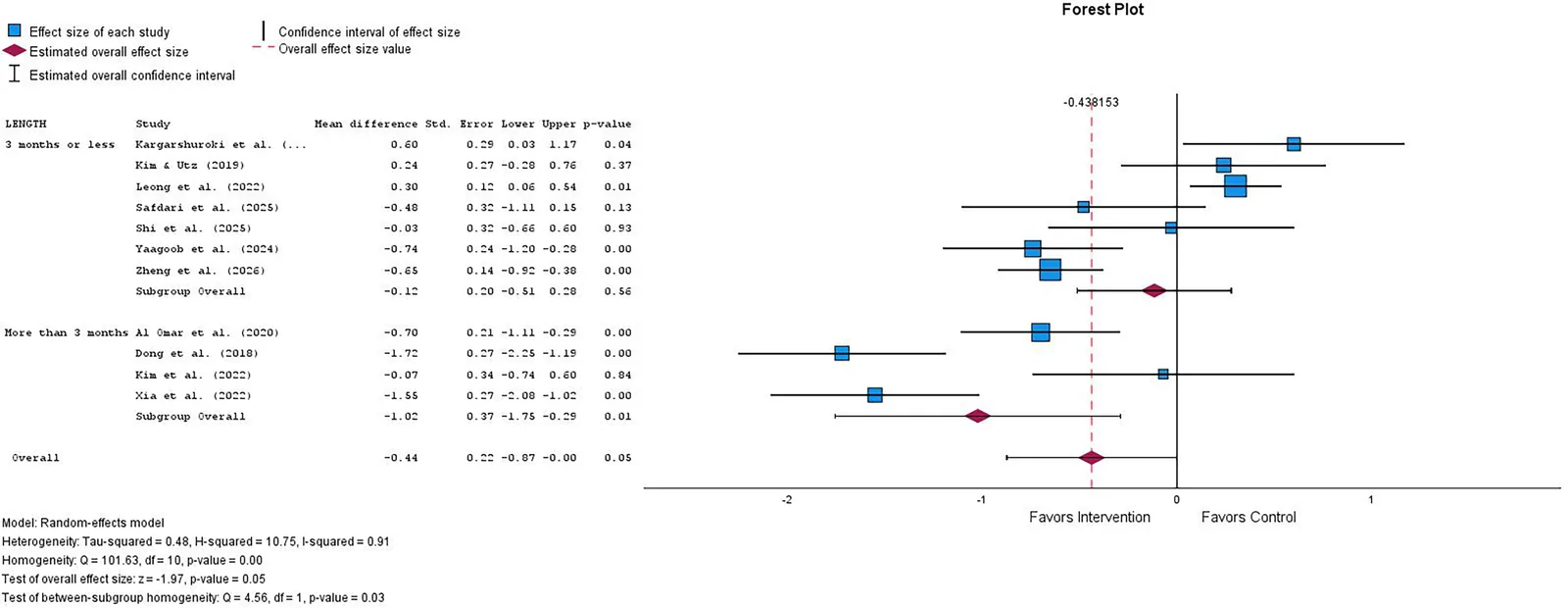
Forest plot of the meta-analysis of HbA1c of the included reviews.

### Subgroup analysis

Given the substantial heterogeneity, an exploratory *post hoc* subgroup analysis was conducted to examine whether intervention duration was associated with differences in effect size. Studies were stratified by intervention duration into two subgroups: ≤ 3 months (*k* = 7) and > 3 months (*k* = 4). For interventions of three months or less, the pooled effect was small and not statistically significant (MD = -0.12, 95% CI -0.51 to 0.28, *p* = 0.56; I^2^ = 84%). In contrast, interventions lasting more than 3 months showed a large, statistically significant effect favoring the intervention (MD = -1.02, 95% CI -1.75 to -0.29, *p* = 0.01; I^2^ = 87%). The test of between-subgroup homogeneity was statistically significant (Q = 4.56; df = 1; *p* = 0.03), indicating that the pooled effect differed by intervention duration. Both subgroups still showed significant heterogeneity, so the duration only explains part of the variability.

### Sensitivity analysis

Leave-one-out sensitivity analyses indicated that the direction of the pooled effect was stable across all 11 iterations, with estimates ranging from -0.31 to -0.54%. However, the pooled estimate reached conventional statistical significance in only four of the 11 iterations, indicating that the overall effect is not robust to removing individual trials. Because

Because the two quasi-experimental studies were excluded from the meta-analysis due to their study design rather than risk of bias, a sensitivity analysis excluding higher-risk studies was not applicable. Consequently, the pooled meta-analysis was based entirely on RCTs assessed as having either low risk of bias or some concerns.

### Use of behavioral theory

Of the 13 studies included, five (38.5%) indicated that they employed a behavioral theory to inform their study interventions. Four of these studies identified SCT as their theoretical framework (47, 52, 53, 65) and one study utilized Bandura’s Self-Efficacy Theory (31), a key component of SCT. The remaining eight studies did not mention a theoretical framework. Notably, social media platforms (such as WeChat, Naver Band, Naver Café, and WhatsApp) can easily facilitate communication, feedback, and participant engagement, elements that align with SCT constructs such as observational learning, reciprocal interaction, reinforcement, and self-efficacy (35, 67, 68).

### Secondary outcomes: self-care, efficacy, and health literacy outcomes

Five studies assessed diabetes self-care (48, 50, 52, 53, 64). Three reported statistically significant improvements in total self-care scores for the intervention groups compared with baseline or control conditions (48, 50, 64).

Three studies assessed self-efficacy (31, 51, 65). All reported statistically significant improvements in self-efficacy post-measurement in those receiving social media-based DSMES, though effect sizes and analytic approaches differed across trials.

Four studies evaluated health literacy adapted to the participants’ language (46, 48, 50, 53). Findings suggested that social media-based interventions may support participants with lower baseline health literacy, with some studies reporting reduced performance gap between lower- and higher-literacy participants.

## Discussion

This systematic review and meta-analysis evaluated the association between social media–based DSMES interventions and HbA1c outcomes among adults with T2DM, with an exploratory *post hoc* analysis to evaluate the potential impact of intervention duration. Across the included studies, participation in social media-based DSMES was generally associated with reductions in HbA1c, and the pooled meta-analysis demonstrated a statistically significant overall effect. While the pooled meta-analytic effect was statistically significant, the substantial between-study heterogeneity (I² = 91%) mandates cautious interpretation.

However, clinical relevance is equally important to consider. The pooled between-group difference was 0.44%, which approaches but does not reach the 0.5% reduction commonly regarded as clinically meaningful in diabetes management and associated with meaningful reductions in the risk of microvascular complications (5–7). Given the substantial heterogeneity across trials, and a prediction interval spanning both benefit and harm, this estimate should be read as an average across diverse interventions rather than an effect expected in any particular setting.

### Sources of heterogeneity

The variability noted across studies (I² = 91%) likely reflects important clinical and methodological differences among the interventions. Studies varied widely in the social media platforms used, including WeChat, WhatsApp, Telegram/Soroush, LINE, Naver Band, and Naver Café. These platforms differ in communication features, program educator capabilities, and potential for peer interaction, all of which may influence intervention effectiveness. Although all studies included principles of DSMES, the interventions varied in their intensity, educational resources, reminders, peer support, self-monitoring, goal setting, and personalized feedback.

Healthcare professional involvement was also diverse. Several interventions were nurse-led or included multidisciplinary diabetes educators providing coaching and monitoring, whereas others primarily used automated educational content or investigator-developed messaging. This variability may partially explain differences in observed treatment effects.

Baseline glycemic control differed considerably across studies, with mean baseline HbA1c ranging from approximately 6.9% to 10.3%. Studies enrolling participants with poorer baseline glycemic control generally demonstrated larger absolute HbA1c reductions, suggesting greater opportunity for improvement. Also, pre- and post-study HbA1c results were captured using direct clinical laboratory testing (53, 60, 62), electronic medical record (EMR) abstraction (30, 58, 59, 61, 66), and point-of-care testing if not in the EMR (55), and participant self-reporting (52).

Geographic variation may also have contributed to heterogeneity. Most studies were conducted in East Asia and the Middle East using culturally tailored interventions delivered in participants’ native languages. Differences in healthcare systems, diabetes education infrastructure, cultural practices, and digital literacy may have influenced implementation and effectiveness.

Therefore, the between-study heterogeneity likely reflects multiple clinical and methodological factors. Exploratory subgroup analyses suggested that interventions lasting longer than three months were associated with larger reductions in HbA1c than interventions of three months or less. These results were also observed in a non-social media meta-analysis of in-person DSME for T2DM, showing that interventions lasting more than six months had the greatest effect (58).

The social media interventions in this synthesis were not limited to younger, tech-savvy individuals; they were commonly implemented among middle-aged and older adults. This has important implications for engagement: adults in their 40s through 60s represent a key demographic for participation in DSMES programs.

On the primary mean difference scale, neither the funnel plot inspection nor Egger’s test indicated asymmetry (p = 0.96), and trim-and-fill analysis imputed no studies. With only 11 heterogeneous trials, these procedures have limited statistical power, so this should not be taken as evidence that publication bias is absent.

The RCTs had a low to some risk of bias, mainly due to inadequate reporting of randomization, which may introduce some uncertainty in the estimates. Conversely, the two quasi-experimental studies faced serious risk of bias from confounding factors and single-arm designs, but their exclusion minimized their effect on the overall estimate.

The search did not yield any English- or Spanish-speaking participant enrollment, highlighting a significant gap in the literature that could be explored in future studies. In the search and data extraction, no eligible non-randomized or quasi-experimental studies of Hispanic populations were identified, further underscoring the scarcity of English-language literature for this demographic. Authors of the U.S. study focused exclusively on Chinese-speaking participants (47) and concluded that the initiative could also serve as a valuable model for other high-risk groups, such as Hispanic populations, who face similar challenges in accessing DSMES and frequently utilize platforms like WhatsApp. The review did not include studies on Hispanic populations; however, existing literature highlights a pressing need for customized digital interventions for these groups as well. Ultimately, modifying DSMES interventions to address factors that influence participation is needed to improve engagement and reduce diabetes- related complications and disparities (69).

From an SCT perspective, social media platforms are well-suited for DSMES (37, 68), including observational learning, peer modeling, social reinforcement, and reciprocal interaction between individuals and their environments. Improvements in self-care behaviors, self-efficacy, and health literacy observed in the studies reviewed further support this theoretical alignment. An integrative review found that health promotion campaigns and studies on social media aimed at changing behavior most often utilized the SCT framework (36).

The findings also highlight the potential of social media–based DSMES to address longstanding barriers to traditional in-person education, including language barriers, limited access, time constraints, workforce shortages, and geographic disparities. This is especially relevant for populations with lower educational attainment and health literacy, which were prevalent across the included studies. Several interventions narrowed disparities between individuals with lower versus higher health literacy, suggesting that social media–based DSMES may promote equity when appropriately designed.

### Limitations

Several limitations should be considered when interpreting the findings of this review and meta-analysis. Although thirteen studies were included, only eleven randomized controlled trials qualified for the meta-analysis, making it relatively small. Second, statistical heterogeneity was across studies was extremely high (I² = 91%). Although a random-effects model, *post hoc* subgroup analyses by duration, and leave-one-out sensitivity analyses were performed, these steps did not fully explain the observed heterogeneity. Consequently, the pooled estimate should be interpreted as an average effect across interventions that differed markedly in platform features, country, language, intervention intensity, educator qualifications, and baseline participant characteristics, rather than as a uniform effect applicable to any particular social-media-based DSMES. The findings may not be generalizable to all adult populations with T2DM.

No randomized controlled trial was deemed to have a high risk of bias; however, most had low or some concerns due to incomplete reporting of randomization. These limitations may affect confidence in the precision of the pooled estimate. Additionally, the small number of available studies made it difficult to analyze the effect of study quality on treatment outcomes.

Additionally, only English-language publications were included, potentially excluding relevant studies in other languages. However, the included studies featured participants’ interventions in Arabic, Chinese, Korean, and Farsi, offering a broader perspective despite language-based publication constraints. The English-language publication restriction may have limited the assessment of within-country research from Latin America, contributing directly to the observed lack of Hispanic populations. The search update conducted in February 2026 was limited to PubMed. Although the initial search of all four databases was comprehensive through December 2025, eligible records published after and not indexed in PubMed may not have been included.

### Implications

Although the pooled findings suggest that social media-based DSMES interventions may achieve clinically meaningful improvements in glycemic control, additional well-designed randomized controlled trials are needed to determine whether these effects are sustained over the long term and which intervention features best support maintenance of health benefits. Future research should evaluate both statistical and clinical significance and identify those components of social media-based DSMES, including platform type, professional healthcare involvement, educational content, peer support, and message frequency, that are associated with improved glycemic outcomes. Standardized reporting of intervention characteristics would facilitate future meta-analyses and improve the transferability and reproducibility of digital DSMES interventions.

## Conclusion

This systematic review and meta-analysis suggest that social media-based DSMES interventions may be associated with reductions in HbA1c and improvements among adults with T2DM. While the pooled meta-analytic effect was statistically significant, the substantial between-study heterogeneity is interpreted with caution. Crucially, this pooled estimate represents an average effect across substantially heterogeneous interventions and populations and therefore should not be interpreted as a uniform effect. Heterogeneity stems from diverse intervention designs, platforms, and populations, highlighting the need for greater standardization and theory-driven interventions in future research. The pooled between-group reduction approaches the 0.5% threshold generally regarded as clinically meaningful, but heterogeneity limits confidence in any single point estimate. Improvements in self-care behaviors, self-efficacy, and health literacy further support the utility of social media platforms as viable and scalable tools for DSMES. Future adequately powered randomized controlled trials utilizing standardized intervention reporting and uniform delivery protocols are essential to evaluate the sustainability of benefits post-intervention. Additionally, researchers should investigate whether engagement mechanisms vary across platforms and assess the potential impact of integrating social media with mobile health tools and primary care services on clinical efficacy and scalability. If supported by strong evidence, well-designed social media DSMES programs could increase access to diabetes education, foster health equity, and enhance long-term diabetes self-management.

## Data Availability

The original contributions presented in the study are included in the article/**Supplementary Material**. Further inquiries can be directed to the corresponding author.
