# Supplementary material for: Effect of social media–based diabetes self-management education and support on glycemic control in adults with type 2 diabetes: a systematic review and meta-analysis
Source: Front Clin Diabetes Healthc. 2026 Oct 28;7:1907185. doi: 10.3389/fcdhc.2026.1907185 (PMC13751530; doi:10.3389/fcdhc.2026.1907185)
Supplement: Supplementary file 1 [file Table1.doc]

| **Identification of Original Term/Keyword/Controlled Vocabulary Based on Search Engines** | | | |  |
| --- | --- | --- | --- | --- |
| **Original Term/**  **Keyword** | **PubMed Equivalent MeSH Term** | **PsycInfo Equivalent Controlled Vocabulary Term** | **Web of Science Complete**  **Controlled Vocabulary Term** | **CINAHL** |
| **Diabetes Self-Management Education OR**  **Diabetes Self Management Education Support OR**  **DSME OR**  **DSMES OR**  **Diabetes Education OR**  **Chronic Disease Education OR**  **Self Management** | "diabetes education"[Title/Abstract] or "diabetes training"[Title/Abstract][Title/Abstract] or "diabetes knowledge" OR DSME[Title/Abstract] OR "Diabetes Mellitus"[Mesh] | TIAB("diabetes education" or "diabetes training" or "diabetes knowledge" OR DSME) OR MAINSUBJECT.EXACT("Diabetes Mellitus") | "diabetes education" or "diabetes training" or "diabetes knowledge" OR DSME | TI("diabetes education" or "diabetes training" or "diabetes knowledge" OR DSME) OR AB(MH "Diabetes Education" OR DSME) OR (MH "Diabetes Education") |
| **Social Media OR Social Networking** | "social media"[Title/Abstract] OR facebook[Title/Abstract] OR twitter[Title/Abstract] OR instagram[Title/Abstract] OR snapchat[Title/Abstract] OR tumblr[Title/Abstract] OR "social networking"[Title/Abstract] OR "Social Media"[Mesh] OR "Social Networking"[Mesh] | TIAB("social media" OR facebook OR twitter OR instagram OR snapchat OR tumblr OR "social networking") OR MAINSUBJECT.EXACT("Social Media") OR MAINSUBJECT.EXACT("Digital Media") OR MAINSUBJECT.EXACT("Online Social Networks") | "social media" OR facebook OR twitter OR instagram OR snapchat OR tumblr OR "social networking" | (MH "Social Media+") OR TI("social media" OR facebook OR twitter OR instagram OR snapchat OR tumblr OR "social networking") OR AB("social media" OR facebook OR twitter OR instagram OR snapchat OR tumblr OR "social networking") |

**PubMed**

| **Concept** | **Keywords** | **Mesh** |
| --- | --- | --- |
| **DSMES** | “Diabetes Self Management Education”[Title/Abstract] OR  “Diabetes Self Management Education Support”[Title/Abstract] OR  “DSME”[Title/Abstract] OR  “DSMES”[Title/Abstract] OR  “Diabetes Education”[Title/Abstract] OR  “Chronic Disease Education”[Title/Abstract] OR  “Self Management”[Title/Abstract] | "diabetes education"[Title/Abstract] or "diabetes training"[Title/Abstract][Title/Abstract] or "diabetes knowledge" OR DSME[Title/Abstract] OR "Diabetes Mellitus"[Mesh] |
| **Social Media** |  | "social media"[Title/Abstract] OR facebook[Title/Abstract] OR twitter[Title/Abstract] OR instagram[Title/Abstract] OR snapchat[Title/Abstract] OR tumblr[Title/Abstract] OR "social networking"[Title/Abstract] OR "Social Media"[Mesh] OR "Social Networking"[Mesh] |

Web of Science

| **Concept** | **Keywords** | **Mesh** |
| --- | --- | --- |
| **DSMES** | “Diabetes Self Management Education”[Title/Abstract] OR  “Diabetes Self Management Education Support”[Title/Abstract] OR  “DSME”[Title/Abstract] OR  “DSMES”[Title/Abstract] OR  “Diabetes Education”[Title/Abstract] OR | "diabetes education" or "diabetes training" or "diabetes knowledge" OR DSME |
| **Social Media** |  | "social media" OR facebook OR twitter OR instagram OR snapchat OR tumblr OR "social networking" |

**CINHAL**

| **Concept** | **Keywords** | **Mesh** |
| --- | --- | --- |
| **DSMES** | TX(“Diabetes Self Management Education” OR  “Diabetes Self Management Education Support” OR  “DSME” OR  “DSMES” OR  “Diabetes Education” OR  “Chronic Disease” “Education” OR  “Self Management”) | TI("diabetes education" or "diabetes training" or "diabetes knowledge" OR DSME) OR AB(MH "Diabetes Education" OR DSME) OR (MH "Diabetes Education") |
| **Social Media** | (MH "Social Media+") OR TI("social media" OR facebook OR twitter OR instagram OR snapchat OR tumblr OR "social networking") OR AB("social media" OR facebook OR twitter OR instagram OR snapchat OR tumblr OR "social networking") | (MH "Social Media+") OR TI("social media" OR facebook OR twitter OR instagram OR snapchat OR tumblr OR "social networking") OR AB("social media" OR facebook OR twitter OR instagram OR snapchat OR tumblr OR "social networking") |

**PsycINFO**

| **Concept** | **Keywords** | **Mesh** |
| --- | --- | --- |
| **Social Media** | TIAB("social media" OR facebook OR twitter OR instagram OR snapchat OR tumblr OR "social networking") OR MAINSUBJECT.EXACT("Social Media") OR MAINSUBJECT.EXACT("Digital Media") OR MAINSUBJECT.EXACT("Online Social Networks") | TIAB("social media" OR facebook OR twitter OR instagram OR snapchat OR tumblr OR "social networking") OR MAINSUBJECT.EXACT("Social Media") OR MAINSUBJECT.EXACT("Digital Media") OR MAINSUBJECT.EXACT("Online Social Networks") |
| **DSMES** | TIAB("diabetes education" or "diabetes training" or "diabetes knowledge" OR DSME) OR MAINSUBJECT.EXACT("Diabetes Mellitus") | TIAB("diabetes education" or "diabetes training" or "diabetes knowledge" OR DSME) OR MAINSUBJECT.EXACT("Diabetes Mellitus") |
